# Measurement invariance and adapted preferences: evidence for the ICECAP-A and WeRFree instruments

**DOI:** 10.1186/s12955-023-02208-9

**Published:** 2023-11-10

**Authors:** Jasper Ubels, Michael Schlander

**Affiliations:** 1 Division of Health Economics, German Cancer Research Center (DKFZ), Heidelberg, Germany; 2 Mannheim Medical Faculty, University of Heidelberg, Mannheim, Germany; 3 Alfred-Weber-Institute for Economics, University of Heidelberg, Heidelberg, Germany

**Keywords:** Adapted preferences, Measurement invariance, Capability approach, Validity, Instrument development, Health economics, Patient-reported outcomes

## Abstract

**Background:**

Self-report instruments are used to evaluate the effect of interventions. However, individuals adapt to adversity. This could result in individuals reporting higher levels of well-being than one would expect. It is possible to test for the influence of adapted preferences on instrument responses using measurement invariance testing. This study conducts such a test with the Wellbeing Related option-Freedom (WeRFree) and ICECAP-A instruments.

**Methods:**

A multi-group confirmatory factor analysis was conducted to iteratively test four increasingly stringent types of measurement invariance: (1) configural invariance, (2) metric invariance, (3) scalar invariance, and (4) residual invariance. Data from the Multi Instrument Comparison study were divided into subsamples that reflect groups of participants that differ by age, gender, education, or health condition. Measurement invariance was assessed with (changes in) the Comparative Fit Index (CFI), Root Mean Square Error of Approximation (RMSEA), and Root Mean Square Residual (SRMR) fit indices.

**Results:**

For the WeRFree instrument, full measurement invariance could be established in the gender and education subsamples. Scalar invariance, but not residual invariance, was established in the health condition and age group subsamples. For the ICECAP-A, full measurement invariance could be established in the gender, education, and age group subsamples. Scalar invariance could be established in the health group subsample.

**Conclusions:**

This study tests the measurement invariance properties of the WeRFree and ICECAP-A instruments. The results indicate that these instruments were scalar invariant in all subsamples, which means that group means can be compared across different subpopulations. We suggest that measurement invariance of capability instruments should routinely be tested with a reference group that does not experience a disadvantage to study whether responses could be affected by adapted preferences.

## Background

Policymakers need reliable information for decision-making. In health policy, this information is partially based on patient-reported outcomes. These outcomes reflect the patients’ experiences of their health condition, which might include an evaluation of how well-off they perceive to be [1]. In this context, adapted preferences could influence responses to instruments [2–4]. Adapted preferences have been defined as follows: “preferences formation or adaptation is the phenomenon whereby the subjective assessment of one’s well-being is out of line with the objective situation” [5, p. 137]. When responding to instruments, patients report a higher level of well-being than one would expect based on their health condition due to these adapted preferences [6, 7]. This is one form of response shift [8].

Differences in the interpretation of items have already been studied for instruments that are used in the wider health economic context [3, 9]. The authors of these studies indicate that such differences in the interpretation of items can affect decision-making when these instruments are used to establish the effect of health interventions [3, 9]. More specifically, the adaptation of preferences by patients might lead to an underestimation of the effect of new health technologies on well-being [10]. To illustrate, if a new health technology improves mobility, it might be difficult to measure its real effect when individuals who adapted to limited mobility report having a high initial level of mobility before the use of such a health technology [10]. This could lead to an unjust allocation of resources if the information that policymakers receive indicates that a new health technology only has a minor effect [10–12].

Adapted preferences might thus affect how individuals interpret and respond to instruments. It is therefore important to test whether different groups interpret and respond to items similarly to ensure that adapted preferences do not affect responses. One way of doing so is by testing for measurement invariance. Measurement invariance has been defined by Millsap [13, p. 462] as follows: “Some properties of a measure should be independent of the characteristics of the person being measured, apart from those characteristics that are the intended focus of the measure”.

Measurement invariance tests have been conducted to study whether instrument responses can be compared across cultures [14, 15], in education to study whether the measured ability of a student can be compared across groups (e.g. [16]), and in psychology to, for example, study if results from personality research can be compared and generalized to various populations [17]. In each of these fields, measurement invariance testing has been used to study whether responses to items are equivalent. This is not only important for research, but could also affect individuals’ lives directly. To illustrate, a mathematics test that is not measurement invariant might penalize certain groups for having a different socioeconomic background, which has little to do with the mathematical ability of a student. Also in the context of quality-of-life instruments measurement invariance testing has been one of the methods to establish whether the interpretation of items and their responses change over time in patient groups [18]. One explanation for this change is that patients adapt to their disease [19]. As such, a measurement invariance test can be a useful tool to study whether patients’ responses are affected by the adaptation of their preferences.

These tests have however not been routinely applied in capability approach inspired instruments in health economics. The capability approach is a theory developed by Sen [20]. Proponents of the capability approach argue that well-being should not only be assessed in terms of what people are or do (also called functionings) but also in terms of their freedom to be or do (capabilities). Based on this theory, several instruments have been developed to assess the impact of health interventions on well-being [21, 22].

Recent reviews of the psychometric properties of these capability instruments did not identify measurement invariance tests [23–25]. Besides these reviews, only one recent publication studied the measurement invariance properties of a capability instrument [26]. Amongst other things, this study tested the measurement invariance properties of the ICECAP-A in different subgroups in a sample of dermatological patients [26]. Measurement invariance could not be established in subgroups where participants were grouped according to age, marital status, or scores on a dermatology-specific quality-of-life index.

We also identified one further qualitative study that aimed to assess whether responses to the ICECAP-A, ICECAP-SCM, and EQ-5D-5 L were influenced by adapted preferences utilizing think-aloud interviews [27]. The authors of this study concluded that there was little indication of adapted preferences in an end-of-life setting [27]. Although this study provides an important insight into this particular group’s reasoning when responding to items, it is unclear if these responses are comparable across groups from a psychometric perspective.

Previous studies in quality of life research have shown that age [4, 28–30], education [31], gender [29], and health condition [30, 32] could affect the interpretation of items. One explanation for these differences is that individuals adapt to adversity [30].

Hence, the primary aim of this study is to establish whether capability instruments can be shown to be measurement invariant across groups of individuals that differ in terms of age, education, gender, or health conditions.

## Methods

### Instruments

The Wellbeing Related option-Freedom instrument (WeRFree) instrument is a newly developed instrument that shows the benefits of developing surveys with a comprehensive conceptualization of the concept of “capability” [33]. The WeRFree instrument consists of 3 scales with a total of 15 items that measure health-related capabilities and subjective well-being [33]. These three scales represent different elements of capability – and subjective well-being. Capability well-being is captured with the “perceived access to options” scale and consists of five items measuring various aspects of health-related capabilities. Different elements of how people experience living with those capabilities are captured with the reflective wellbeing (six items) and affective wellbeing (four items) scales. All items follow a Likert scale format, with response options ranging from four to eleven categories. Depending on the construct, items inquire about the extent that individuals feel satisfied with various aspects of their lives (from completely dissatisfied to completely satisfied), whether they disagree with certain statements (from strongly disagree to strongly agree), whether they experienced certain emotions over the last four weeks (e.g. from all of the time to none of the time), and whether individuals can complete certain tasks (e.g. whether an individual can do tasks very quickly and efficiently without any help to not being able to do these tasks themselves). The WeRFree instrument was developed by matching items from the Multi-Instrument-Comparison (MIC) study database with constructs from an earlier developed theoretical framework by the authors [33, 34]. Further information about the (theoretical) background of the instrument can be found in [22, 33, 34].

The ICEpop CAPability measure for Adults (ICECAP-A) is an instrument that was developed to assess the capability well-being of adults [35, 36]. The ICECAP-A measures capabilities in five domains: stability, attachment, autonomy, achievement, and enjoyment. Each of these domains consists of a single item, with each item having four response options. Each item inquires about the level of capability, ranging from no capability (I cannot…, I am unable…) to full capability (I can…, I am able to…). Together, these items reflect the capability well-being of individuals. The domains and items were developed through interviews with the general population of England [35]. Evidence indicates that the instrument shows construct validity, content validity and responsiveness in a number of different populations [25]. 

### Data

For this study, the MIC study database was used [37]. The MIC study had the objective to analyze and compare a set of HRQoL and well-being instruments. The general questionnaire of this study consisted of eleven such instruments. Following a cross-sectional design, the study was conducted in six countries: Australia, Canada, Germany, Norway, the United Kingdom, and the USA. A total of 9665 respondents participated in completing the general questionnaire. Informed consent was obtained from all individual participants included in the study. Individuals were recruited with nine different health conditions: arthritis, asthma, cancer, depression, diabetes, hearing problems, heart problems, stroke, and obstructive pulmonary disease. Additionally, a group of healthy individuals was recruited. Unreliable responses were removed from the database by the MIC study team. Responses were deemed unreliable if they showed inconsistencies in responses (i.e. between items that are similar) and if respondents took too little time to complete the general questionnaire. After the removal of these responses, the MIC study database consisted of 8022 observations. Further information about the MIC study can be found on the website of the project [38]. Concerning the analysis of the ICECAP-A, all the responses of the MIC database were used, except those from Norway, since in Norway the ICECAP-A instrument was not administered. For measurement invariance testing, different subsamples were created based on the characteristics of the participants. Participants were grouped according to their age, level of education, gender, and health condition. Measurement invariance was then tested in each of these subsamples with the WeRFree and ICECAP-A instruments.

### Analyses

Before conducting a measurement invariance study, the dimensionality of instruments needs to be studied. This was done through a confirmatory factor analysis (CFA). Model fit was considered acceptable when the following fit index values reached certain values: Comparative Fit Index (CFI) with a value higher than 0.900, Tucker-Lewis Index (TLI) with a value higher than 0.900, Root Mean Square Error of Approximation (RMSEA) with a value lower than 0.08, and Standardized Root Mean Square Residual (SRMR), with a value lower than 0.08 [39–41]. The model fit of the WeRFree instrument with the MIC data has been presented in an earlier study that further explains how the instrument was developed [33]. In the case of the ICECAP-A, we followed the approach of Rencz, Mitev [26] and conducted a CFA to study the dimensionality of the ICECAP-A, for which we assumed that the five items reflect one construct: capability wellbeing. Additionally, the Cronbach’s alpha was computed, with a cut-off value of > 0.7 deemed acceptable. 

A multi-group CFA was conducted to test for four different types of measurement invariance: (1) configural invariance, (2) metric (or weak factorial) invariance, (3) scalar (or strong factorial) invariance, and (4) residual (or strict) invariance [41–44]. These types were tested sequentially since for each type of measurement invariance a different model is constructed that is more restrained than the last model.

An instrument is (1) configural invariant if its factorial structure can be reproduced in different groups. In the case of the current study, this would for example mean that the three-factor structure of the WeRFree instrument can be replicated in different groups. When configural invariance can be established, (2) metric invariance can be tested [41, 42]. An instrument is metric invariant when the factor loadings are invariant across different groups. The factor loading represents the strength of the relationship between a construct and an item, or, in other words, how far a change in a construct influences the response to an item from an individual. Invariant factor loadings indicate that the constructs influence changes in item scores in the same way in different groups. The third type of invariance that is tested for in this study is (3) scalar invariance. An instrument is scalar invariant when the intercepts of each item are the same across different health conditions. Once scalar invariance is established, it is possible to compare the mean scores of the scales between different groups [41, 42]. Lastly, the (4) residual invariance properties were studied. Essentially, this means that the residuals of the items are similar across different groups. This indicates that the mean differences in scale scores that can be observed between groups are a result of differences in the latent construct and are not caused by other factors [41, 42]. This provides additional confidence that the difference in mean scores is indeed driven by differences in the latent construct of interest and not by other unmeasured constructs [41, 42].

In the current analysis, for both the WeRFree instrument and the ICECAP-A, mean factor scores will be presented. Furthermore, for the WeRFree instrument, adjusted scale scores are presented. Due to the varying number of response categories of the items, scale scores were normalized by dividing the number of response categories of items by their respective length (e.g. an item with a score from 0 to 3 was divided by 3), multiplying that score by 100, and dividing that score by the number of items in a scale to ensure that the score of each item contributed equally to the overall score of scale. Also ICECAP-A scores are presented, with raw index values being adjusted according to the United Kingdom tariff developed by Flynn, Huynh [36]. This score ranges from zero to one, with a zero reflecting a state of no capability and a one a state of full capability [36].

Various fit indices were used to establish measurement invariance. The following fit index values were used to establish configural fit: CFI with a value higher than 0.900, RMSEA with a value lower than 0.08, and SRMR with a value lower than 0.08 [39–41]. To study the other forms of measurement invariance, we followed the suggested fit index values by Chen [41] for group sizes that are equal to or larger than 300, because the sample sizes of the groups in the different subsamples are larger than 300. For further measurement invariance testing, the ΔCFI, the ΔRMSEA, and the ΔSRMR fit indices were used. A score of ≥ 0.010 in ΔCFI, ≥ 0.015 in ΔRMSEA, and a score of ≥ 0.030 in SRMR indicated noninvariance regarding metric invariance. Scores of ≥-0.010 in ΔCFI, ≥ 0.015 in ΔRMSEA, and ≥ 0.010 in SRMR were used as an indication of noninvariance regarding scalar and residual invariance. The chi-square difference test was not used to assess and compare model fit, because of the large sample sizes of the subsamples, which would result in trivial differences in model fit being flagged as significant [41].

For the analysis presented in this manuscript, the Lavaan package was used in R [45]. Because some response options of some of the items included in this study received close to no responses, it was decided not to use polychoric correlations, since in such cases correlations could be estimated incorrectly, which affects the estimation of parameters of CFA models [46]. Instead, Pearson correlations were used for model estimation, given that the sample sizes in each group were reasonably large (the smallest group had more than 500 observations, see Table 1) and that the number of response options for the items was generally larger than five. In such conditions, authors have argued that data can be treated as continuous [47, 48]. For the same reasons, it was decided to estimate the models with a maximum likelihood estimator [47, 49]. In these estimates, missing data were handled through a full information maximum likelihood estimation of the models [50].

## Results

### Data

Table 1 presents the sample size per subsample, as well as the size of different groups within those subsamples. It should be noted that the total size of the health condition subsample is slightly lower compared to the size of the other subsamples. This is a consequence of the deletion of two “artifact” disease groups. During the recruitment phase of the MIC study project, the Australian arm also recruited patients affected by stroke and chronic obstructive pulmonary disease. These subgroups consisted of 23 and 66 participants respectively. The sample sizes of these groups were considered to be inadequate for further analysis and the observations were not included for measurement invariance testing in the health condition subsample. Furthermore, 15 observations in the MIC database showed missing data concerning the items included on the “Reflective Wellbeing” scale of the WeRFree instrument.

**Table 1:** Sample size per group

Subsample	WeRFree instrument *n* (%)	ICECAP-A^a^ *n* (%)
Age group subsample		
18–24	513 (6.39%)	421 (6.15%)
25–34	944 (11.77%)	825 (12.05%)
35–44	1137 (14.17%)	998 (14.58%)
45–54	1689 (21.05%)	1487 (21.72%)
55–64	2008 (25.03%)	1732 (25.30%)
65+	1731 (21.58%)	1382 (20.19%)
Gender subsample		
Men	3848 (47.97%)	3138 (45.84%)
Women	4174 (52.03%)	3707 (54.16%)
Education subsample		
High school	2522 (31.44%)	2193 (32.04%)
Some post-secondary, post-secondary certificate or diploma	3241 (40.40%)	2670 (39.01%)
University degree and higher	2259 (28.16%)	1982 (28.96%)
Total in age group, gender, and education subsamples	8022 (100%)	6845 (100%)
Health condition subsample ^b^		
Healthy public	1760 (22.19%)	1472 (21.79%)
Arthritis	929 (11.71%)	799 (11.82%)
Health condition subsample^b^		
Asthma	856 (10.79%)	726 (10.74%)
Cancer	772 (9.73%)	692 (10.24%)
Depression	917 (11.56%)	777 (11.17%)
Diabetes	924 (11.65%)	781 (11.56%)
Hearing problems	832 (10.49%)	717 (10.61%)
Heart problems	943 (11.89%)	792 (11.72%)
Total in health condition subsample	7933 (100%)	6756 (100%)

^a^ Respondents from Norway did not complete the ICECAP-A.

^b^ Respondents affected by stroke or obstructive pulmonary disease were removed from the analysis in the health condition subsample, with a reduced sample size in the health condition subsample as a result.

### WeRFree instrument

As mentioned in the methods section, the WeRFree instrument has shown an adequate fit with the MIC data (χ2: 1,756.8, df: 87, CFI: 0.970, TLI: 0.963, RMSEA: 0.055, SRMR: 0.036, see Ubels, Hernandez-Villafuerte [33]). Also, the three scales of the WeRFree instrument showed adequate reliability (Perceived Access to Options: Cronbach’s alpha of 0.89, Affective Wellbeing: Cronbach’s alpha of 0.83, Reflective Wellbeing: Cronbach’s alpha of 0.89, see Ubels, Hernandez-Villafuerte [33]). The results of the measurement invariance tests are presented in Table 2. Configural invariance was established in every subsample: the highest value for the upper level of the RMSEA 90% confidence interval was reached in the health condition and age group subsamples with a value of 0.060, the highest SRMR value is 0.041 in the health condition subsample, and the lowest CFI value being 0.961 in the health condition subsample. Metric invariance was also established in every subsample. The largest reduction in model fit in terms of CFI and SRMR could be identified in the health condition subsample, with a reduction of 0.003 and 0.008 respectively. Scalar invariance was also established in every subsample. The largest reductions in RMSEA and SRMR, 0.004 and 0.004 respectively, were identified in the age groups subsample, furthermore, a 0.010 (rounded up) reduction in CFI was identified in the health condition subsample. Residual invariance was not established in the age group and health condition subsamples. To conclude, the WeRFree instrument was measurement invariant up to scalar invariance in the health condition and age group subsamples. Full measurement invariance was established in the gender and education subsamples. Table 3 presents the mean scale scores with the associated standard deviations, as well as the standardized factor means per subsample for the constructs of the WeRFree instrument.

**Table 2:** Measurement invariance of the WeRFree instrument per subsample

Subsample	Model	Χ^2^ (*df*)	CFI	RMSEA (90% CI)	SRMR	Δ Χ^2^ (Δ *df*)	Δ CFI	Δ RMSEA	Δ SRMR
Health condition	Configural invariance	2990.14 (696)	0.961	0.058 (0.056–0.060)	0.041	-	-	-	-
	Metric invariance	3257.81 (780)	0.958	0.057 (0.055–0.059)	0.049	267.67 (84)	-0.003	-0.001	0.008
	Scalar invariance	3916.75 (864)	0.948	0.060 (0.058–0.062)	0.052	658.93 (84)	-0.010	0.003	0.003
	Residual invariance	7233.82 (969)	0.893	0.081 (0.079–0.082)	0.081	3317.08 (105)	-0.055	0.021	0.029
Age	Configural invariance	2853.83 (522)	0.966	0.058 (0.056–0.060)	0.039	-	-	-	-
	Metric invariance	3004.33 (582)	0.965	0.056 (0.054–0.058)	0.043	150.50 (60)	-0.001	-0.002	0.004
	Scalar invariance	3669.88 (642)	0.956	0.059 (0.057–0.061)	0.047	665.54 (60)	-0.009	0.004	0.004
	Residual invariance	4465.45 (717)	0.945	0.062 (0.061–0.064)	0.051	795.58 (75)	-0.011	0.003	0.004
Gender	Configural invariance	2367.76 (174)	0.969	0.056 (0.054–0.058)	0.036	-	-	-	-
	Metric invariance	2399.61 (186)	0.969	0.054 (0.052–0.056)	0.037	31.84 (12)	-0.000	-0.002	0.001
	Scalar invariance	2705.98 (198)	0.965	0.056 (0.054–0.058)	0.039	306.37 (12)	-0.004	0.002	0.002
	Residual invariance	3028.31 (213)	0.960	0.057 (0.056–0.059)	0.041	322.33 (15)	-0.004	0.001	0.002
Education	Configural invariance	2498.27 (261)	0.968	0.053 (0.055–0.059)	0.037	-	-	-	-
	Metric invariance	2565.40 (285)	0.968	0.055 (0.053–0.057)	0.039	67.13 (24)	-0.001	-0.002	0.002
	Scalar invariance	2678.03 (309)	0.966	0.054 (0.052–0.055)	0.040	112.63 (24)	-0.001	-0.001	0.001
	Residual invariance	3236.31 (339)	0.959	0.057 (0.055–0.058)	0.042	558.29 (30)	-0.008	0.003	0.002

Chi-score (Χ), Comparative Fit Index (CFI), Degrees of freedom (df), Root Mean Squared Error of Approximation (RMSEA) with 90% Confidence Intervals (CI), Standardized Root Mean Residual (SRMR).

**Table 3:** Mean scale scores, associated standard deviations and standardized mean factor scores per subgroup per sample for the WeRFree instrument

Subsample	Reflective wellbeing			Affective wellbeing			Perceived access to options		
Age	Mean	Standard deviation	Factor mean*	Mean	Standard deviation	Factor Mean*	Mean	Standard deviation	Factor mean*
18–24	62.01	19.01	Reference	63.61	20.23	Reference	87.51	14.82	Reference
25–34	62.57	19.50	0.000	64.04	19.46	0.012	84.90	16.89	-0.159
35–44	61.51	20.78	-0.061	64.32	20.64	0.006	80.63	19.94	-0.348
45–54	61.68	20.62	-0.059	66.46	21.08	0.101	77.07	21.22	-0.493
55–64	65.18	19.78	0.127	70.71	20.05	0.330	76.92	21.31	-0.514
65+	72.09	16.48	0.598	78.54	15.67	0.996	81.25	18.93	-0.340
Gender									
Women	65.15	20.08	Reference	67.18	20.40	Reference	78.82	20.44	Reference
Men	64.64	19.43	-0.023	71.72	19.68	0.276	81.33	19.45	0.131
Education									
High school	62.80	20.72	Reference	67.49	21.20	Reference	79.39	20.99	Reference
Some post-secondary, post-secondary certificate or diploma	64.93	19.49	0.117	69.52	20.03	0.118	79.40	20.01	0.084
University degree and higher	67.21	18.83	0.263	71.22	19.04	0.239	83.14	18.48	0.297
Health condition									
Healthy public	71.17	15.99	Reference	77.70	14.27	Reference	93.01	9.27	Reference
Arthritis	66.10	19.18	-0.296	70.95	18.47	-0.416	70.63	20.51	-1.127
Asthma	65.34	18.24	-0.340	68.77	17.98	-0.541	82.13	18.41	-0.592
Cancer	65.39	19.53	-0.329	69.92	19.43	-0.435	75.44	20.93	-0.851
Depression	48.72	20.76	-1.167	46.31	20.19	-1.782	70.61	20.96	-1.112
Diabetes	63.04	20.70	-0.421	68.82	20.14	-0.488	77.04	21.08	-0.769
Hearing problems	68.72	18.21	-0.148	74.10	16.65	-0.245	85.05	15.54	-0.501
Heart problems	65.74	18.98	-0.318	71.60	19.64	-0.345	76.59	21.09	-0.794

* The presented factor means are standardized.

### ICECAP-A

**Fig. 1:**
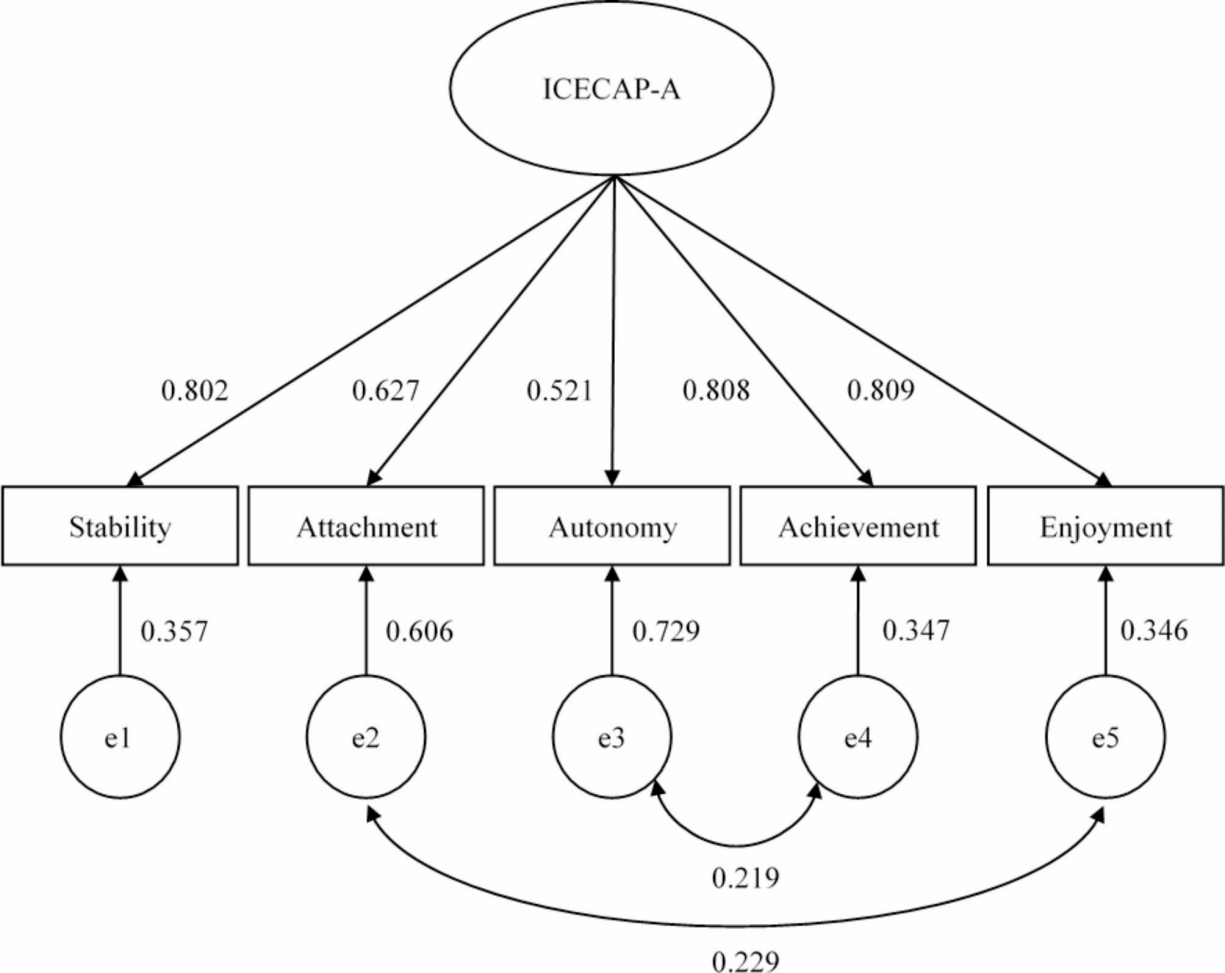
Measurement model of the ICECAP-A with standardized parameter values

The initial model, in which all of the items of the ICECAP loaded on one factor, showed inadequate fit in terms of the RMSEA index value (CFI = 0.961, TLI = 0.922, RMSEA = 0.129, SRMR = 0.033). Upon inspecting the modification indices, we found that two pairs of items showed local dependencies: the items related to attachment and enjoyment (expected improvement in ΔΧ^2^ of 329, expected standardized correlation of 0.281), and the items related to autonomy and achievement (expected improvement in ΔΧ^2^ of 320, expected standardized correlation of 0.318). Due to the small difference in the change in ΔΧ^2^, and the fact that the next two largest sources of misfit were also associated with the attachment item (expected improvement in ΔΧ^2^ of 179 when correlated with the achievement item and an expected improvement in ΔΧ^2^ of 145 when correlated with the autonomy item), we decided to first correlate the attachment and the enjoyment items. Still, the RMSEA indicated inadequate fit (CFI = 0.982, TLI = 0.955, RMSEA = 0.099, SRMR = 0.033). Therefore, we decided to correlate the error terms of the autonomy and achievement items, which resulted in an adequate fit (CFI = 0.995, TLI = 0.982, RMSEA = 0.062, SRMR = 0.013). This resulted in the measurement model presented in Fig. 1, which also presents standardized values for various parameters. The Cronbach’s alpha of the ICECAP-A with the complete sample of the MIC study database is 0.85.

Table 4 presents the results of the measurement invariance test of the ICECAP-A instrument. Configural invariance was established in every subsample: the highest value for the upper level of the RMSEA 90% confidence interval was reached in the age group subsamples with a value of 0.078. In terms of SRMR, the highest value is 0.013 in the age group subsample. The CFI scores were generally very high, around 0.995 in every subsample. Metric invariance of the ICECAP-A was also established in every subsample. In terms of RMSEA, the model fit improved in every subsample. A particular large negative change in terms of SRMR could be identified in the health condition subsample, with a change of 0.017. Scalar invariance was also established in every subsample, although borderline in the age group subsamples in terms of RMSEA (ΔRMSEA = 0.010 rounded). The CFI values of the age group and health condition subsample changed by 0.009 and 0.008 respectively. Residual invariance could not be established for the health condition subsample (ΔCFI = -0.026, ΔRMSEA = 0.018, ΔSRMR = 0.024). The other subsamples were residual invariant. This means that for the ICECAP-A, full measurement invariance has been established in the age group, gender, and education subsamples. Table 5 presents the adjusted scores of the ICECAP-A with associated standard deviations, as well as mean factor scores.

**Table 4:** Measurement invariance of the ICECAP-A per subsample

Subsample	Model	Χ^2^ (*df*)	CFI	RMSEA (90% CI)	SRMR	Δ Χ^2^ (Δ *df*)	Δ CFI	Δ RMSEA	Δ SRMR
Health condition	Configural invariance	100.77 (24)	0.994	0.062 (0.049–0.074)	0.013				
	Metric invariance	164.46 (52)	0.991	0.051 (0.042–0.059)	0.031	63.69 (28)	-0.003	-0.011	0.017
	Scalar invariance	293.06 (80)	0.983	0.056 (0.049–0.063)	0.039	128.60 (28)	-0.008	0.006	0.008
	Residual invariance	652.75 (115)	0.957	0.074 (0.069–0.080)	0.063	359.69 (35)	-0.026	0.018	0.024
Age	Configural invariance	107.51 (18)	0.994	0.066 (0.054–0.078)	0.014				
	Metric invariance	154.11 (38)	0.992	0.052 (0.043–0.060)	0.028	41.33 (20)	-0.002	-0.014	0.014
	Scalar invariance	310.23 (58)	0.982	0.062 (0.055–0.069)	0.034	159.37 (20)	-0.009	0.010	0.007
	Residual invariance	431.51 (83)	0.976	0.061 (0.055–0.066)	0.038	124.29 (25)	-0.007	-0.001	0.004
Gender	Configural invariance	85.82 (6)	0.995	0.062 (0.051–0.074)	0.012				
	Metric invariance	90.45 (10)	0.995	0.048 (0.040–0.058)	0.015	4.63 (4)	0.000	-0.014	0.002
	Scalar invariance	162.36 (14)	0.990	0.056 (0.048–0.063)	0.022	71.91 (4)	-0.005	0.007	0.008
	Residual invariance	182.10 (19)	0.989	0.050 (0.044–0.057)	0.025	19.74 (5)	-0.001	-0.006	0.003
Education	Configural invariance	92.66 (9)	0.994	0.064 (0.052–0.076)	0.013				
	Metric invariance	100.89 (17)	0.994	0.047 (0.038–0.055)	0.017	8.23 (8)	-0.000	-0.017	0.004
	Scalar invariance	137.35 (25)	0.992	0.044 (0.037–0.052)	0.020	36.45 (8)	-0.002	-0.002	0.003
	Residual invariance	186.80 (35)	0.990	0.044 (0.038–0.050)	0.025	49.46 (10)	-0.003	-0.001	0.005

Chi-score (Χ), Comparative Fit Index (CFI), Degrees of freedom (df), Root Mean Squared Error of Approximation (RMSEA) with 90% Confidence Intervals (CI), Standardized Root Mean Residual (SRMR).

**Table 5:** ICECAP-A scores and associated standard deviation per group

Subsample	ICECAP-A		
Age	Mean score*	Standard deviation*	Factor mean**
18–24	0.82	0.18	Reference
25–34	0.80	0.19	-0.142
35–44	0.78	0.19	-0.281
45–54	0.78	0.20	-0.289
55–64	0.81	0.18	-0.153
65+	0.87	0.14	0.241
Gender			
Women	0.80	0.19	Reference
Men	0.82	0.18	0.106
Education			
High school	0.79	0.20	Reference
Some post-secondary, post-secondary certificate or diploma	0.81	0.18	0.074
University degree and higher	0.83	0.17	0.237
Health condition			
Healthy public	0.89	0.12	Reference
Arthritis	0.81	0.17	-0.528
Asthma	0.82	0.17	-0.588
Cancer	0.81	0.18	-1.584
Depression	0.63	0.22	-0.563
Diabetes	0.80	0.19	-0.304
Hearing problems	0.85	0.15	-0.603
Heart problems	0.81	0.18	-0.490

*Adjusted scores. For the adjustment, the tariff developed by Flynn, Huynh [36] was used. This adjusted score ranges from 0 to 1.

**Factor means are standardized.

## Discussion

In this study, the measurement invariance properties of the WeRFree and the ICECAP-A instruments were tested. Before testing the measurement invariance properties of the ICECAP-A, it was necessary to adjust its measurement model by correlating two error terms, because the one factor model without error terms indicated insufficient fit. Given that these adjustments were data-driven, only post-hoc explanations can be provided for why these items might correlate. In the case of the attachment and enjoyment items, it could be that there is an additional correlation between these items due to the strong relationship between social relations and happiness. The errors of these items were also correlated in a previous study by Rencz and Mitev [26]. Such a relationship might also exist for the achievement and autonomy items, since experiences of independence and progress could be closely related to each other and might exhibit correlations that are not explained by the overall latent variable of capability wellbeing. These relationships could be an interesting subject for future confirmatory studies.

In the current study, the instruments were shown to have configural, metric, and scalar invariant properties in the tested subsamples. The establishment of scalar invariance in every subsample indicates that the instruments’ mean scores can be compared on a group level. By comparing the responses of individuals who are relatively disadvantaged in terms of their capabilities (e.g. due to disease) with a reference group (e.g. healthy individuals), it is possible to establish whether responses are affected by adapted preferences. Such reference groups have also been used, albeit not routinely, to test for response shift in patient responses [18, 19].

In the context of testing for the measurement invariance properties of capability instruments in populations that differ in terms of their health condition, the identification of a reference group might be a challenge. Such a reference group should have a set of capabilities that ensures that adapted preferences do not affect the responses of this reference group. However, what such a set entails or how such a list should be constructed is not clear [51], which complicates the identification of a reference group. In this context, more research is necessary. For the time being, it might be sufficient to use a sample from the general population that is reasonably healthy to test for adapted preferences in individuals with health problems.

As was mentioned in the introduction, testing for measurement invariance could indicate how adapted preferences affect responses to instruments. In this context, it is important to note that establishing measurement invariance between advantaged and disadvantaged groups is not evidence against the existence of adapted preferences. As noted, measurement invariance testing merely tests whether response patterns of items differ between different groups. Systematic differences in how individuals respond to instruments between groups, such as overall response styles, are hard to detect with such tests [52]. Furthermore, if measurement invariance cannot be established, it should be noted that the source of measurement noninvariance does not necessarily need to be adapted preferences, since there can be several alternative explanations for why individuals interpret items differently. Lastly, it should be noted that depending on the research aim, different levels of measurement invariance might be sufficient. For example, for studying the correlations between constructs, it is sufficient to establish configural invariance. If a study aims to research a change in a construct of interest, which is often the case in health economics, it is sufficient to establish metric variance.

When measurement invariance cannot be established, further studies can be conducted to identify the source of measurement non-invariance [44, 53]. It should however be noted that establishing non-invariance does not mean that groups cannot meaningfully be compared. Indeed, it can be the case that the non-invariance of items is symmetrically distributed, which means that the non-invariance of multiple items has little effect on the scale score [52]. As such, it is important to study the pattern of non-invariance [54]. Studying these patterns could also lead into interesting insights in how items are interpreted and responded to [54], which could further result in deeper insights in how people experience their capability well-being.

### Limitations

The recruitment strategy of the MIC study aimed at recruiting a sufficient number of participants from different health backgrounds for their database that gave reliable responses [38]. As such, the database was not necessarily designed to reflect specific (sub-) populations. Therefore, the measurement invariance test results as well as the comparison of scale scores and factor means should not directly be generalized. A further limitation is that in the current analysis, the overall sample is divided into different subsamples based on variables that are probably not independent from each other. This affects the interpretation of non-invariance test results, since it is unclear what the exact source of residual noninvariance is. For example, in the case of the WeRFree instrument, residual invariance could not be established in the health condition and age group subsamples. In this case, it is unclear if age, the health condition, or an interaction between age and health condition could explain why this invariance exists. Given that the MIC study sample was not meant to reflect specific populations, we decided to not test in detail what the source of noninvariance was, since the result of such a test would only have limited generalizability.

Another limitation concerns the use of the MIC study database to both develop an instrument and test the measurement invariance properties of the WeRFree instrument. Due to using the same database for both these studies, measurement errors that can be attributed to the design of the MIC survey may be unaccounted for. As a consequence, the measurement models might overfit, which in the context of the present study means that the measurement invariance properties of the WeRFree instrument can be overestimated.

### Conclusion

To conclude, this study shows how measurement invariance testing can be used to research whether adapted preferences influence instrument responses. The study shows that the WeRFree and ICECAP-A instruments are at least scalar invariant in various subpopulations of the MIC study. This indicates that aggregated responses can be compared across different groups. However, due to the limitations of this study, this result needs to be confirmed in other samples. In the context of capability instrument development, future studies should focus on establishing the measurement invariance properties of these instruments. This would clarify whether information from self-report capability instruments is comparable across groups that differ in terms of their relative advantage.

## Data Availability

The data that support the findings of this study are not publicly available. Data can however be requested by contacting the researchers that are responsible for the Multi Instrument Comparison Study.
